# Medicine and the Empire

**Published:** 1912-07-20

**Authors:** 


### The Hospital

The Workers' Newspaper of Administrative Medicine and Institutional Life, Administration, National Insurance and Health.

No. 1359, Vol. LII. SATURDAY, JULY 20, 1912. PRICE ONE PENNY.

### MEDICINE AND THE EMPIRE.

The Secretary of State for the Colonies, in giving what should in future prove a valuable annual résumé of the work done under the auspices of his department during the past year, referred pointedly to the part played by medical men and scientific investigators in Imperial affairs. The fact is often overlooked that no European country could be a great colonising power in tropical parts were it not for the knowledge derived from experimental and clinical research, and the practical application of principles learned by patient and persevering study of abnormal conditions. The use of quinine made possible the ingress of the white man into tropical territories in days when the refinements of modern science were not yet available. The prophylactic use of wire netting, the inculcation of the rules of tropical hygiene and sanitation, both at home and in the Colonies, and the discovery of the ways in which some tropical diseases are spread have done much to help the white man to keep up his standing and prestige in the dependencies over sea which lie within the limits of the equatorial or sub-tropical zone.

But it is evident that medical science has as yet done only a relatively small share of the work that lies before it in connection with the preservation of human life and health in the tropics. Laymen who know something about the mosquito theory of malaria, and who are acquainted with the vast progressive strides that attention to sanitation has brought about in various Colonies, may be pardoned if, like Mr. Harcourt, they take a very rosy view of the advances and optimistically consider that medical science in this matter has all the kudos and nothing in the way of failure and defeat to chronicle. This is, of course, far from being the case. Malta fever, said to be " exterminated " in the island, is by no means eradicated yet; it flourishes among the Maltese in districts where sanitation and hygienic improvement have not been attended to. Mr. Harcourt told his hearers in Parliament that the cause of beri-beri has at last been discovered. It is a claim that has been made from time to time since 1876, when the ravages of the disease among the army in Atjeh attracted general scientific attention to the subject. We may, therefore, be sceptical regarding the value of the new etiological factor which is said to have been discovered, especially when we find that it is our old friend rice. The rice hypothesis is one of the oldest, but, notwithstanding its comparative antiquity, it has yet to be proved. Conditions simulating beri-beri in animals and induced by experimental feeding are comparatively easy to produce, but there exists a justifiable difference of opinion as to whether these conditions can rightly be called beri-beri or whether they are conditions of myelitis produced by intoxication. Our own opinion inclines to favour the latter view, and we have seen or heard nothing to justify the optimistic statements of the Colonial Secretary which tend to lead the layman to suppose that this frightful disease, the scourge of the Eastern labour colonies, is conquered by science, and that we possess a sure and certain safeguard against it. On the contrary, beri-beri is still a puzzle to medical science. It is probable that it is not an intoxication but an infectious disease, as is claimed by the Dutch doctors, who, more than any other investigators, have done excellent work in clearing up the hidden facts of incidence and symptomatology.

Mr. Harcourt is still more impressive to the layman when he lays down what may be taken to be guiding lines in the future. Doubtless Colonial policy must to some extent depend on the advance in tropical medicine. But is it wise to accept as the basis of such policy hypotheses (not theories, as Mr. Harcourt calls them) of etiology and causation which are as yet merely suggestive? Those who believe that the germs of sleeping sickness are spread by game through the medium of the tsetse fly favour the destruction of all big game in the affected regions. Formerly it was proposed to slaughter all the crocodiles; now it is proposed to stamp out all the buck; in a couple of years, when we have discovered that the trypanosome may be carried by bird parasites, by insect parasites, and perhaps also by fruit, there will probably be an appeal to the Government to eradicate all these bearers by a policy which, if carried out to the bitter end, will leave Africa birdless, gameless, and fruitless. Such portentous developments can only be tried on a comparatively small scale, and then only after the suggestive hypotheses have been carefully investigated by an international committee of scientific men. Otherwise there is danger of overlooking the wisest adage in Colonial government—that which inculcates on the Government the duty of hastening slowly. Tropical medicine has been and will still be a good helper, but it is a bad master, especially when it is fashioned and moulded by laboratory workers who disregard local conditions, native customs and traditions, and build upon experimental findings alone.

